# Acute pancreatitis as a complication of childhood cancer treatment

**DOI:** 10.1002/cam4.649

**Published:** 2016-02-13

**Authors:** Milica Stefanović, Janez Jazbec, Fredrik Lindgren, Milutin Bulajić, Matthias Löhr

**Affiliations:** ^1^ Division of PediatricsUnit of Hemato‐oncologyUniversity Medical Centre LjubljanaLjubljanaSlovenia; ^2^ Department of PediatricKarolinska University HospitalStockholmSweden; ^3^ Department of GastroenterologyUniversity Hospital Center “Santa Maria della Misericordia”UdineItaly; ^4^ Faculty of MedicineUniversity of BelgradeBelgradeSerbia; ^5^ Department of Clinical Science, Intervention and TechnologyKarolinska InstitutetStockholmSweden

**Keywords:** Acute pancreatitis, childhood cancer, L‐asparaginase, diagnosis, management

## Abstract

Acute pancreatitis (AP) is now well recognized as a possible complication of childhood cancer treatment, interrupting the chemotherapy regimen, and requiring prolonged hospitalization, possibly with intensive care and surgical intervention, thereby compromising the effect of chemotherapy and the remission of the underlying malignant disease. This review summarizes the current literature and presents the various etiological factors for AP during chemotherapy as well as modern trends in the diagnosis and therapy of AP in children.

## Introduction

Acute pancreatitis (AP) is defined as the histological inflammation of the pancreatic parenchyma, presenting clinically as a sudden onset of abdominal and back pain accompanied by the elevation of pancreatic enzymes in the blood and urine 1, 2. It is a reversible process characterized by the presence of inflammatory cells and varying degrees of cellular apoptosis, necrosis, and hemorrhage 3. AP is seen relatively rarely in children in whom the underlying etiologies differ greatly from those in adults 4. However, recent reports in the literature have confirmed an increasing number of patients with pancreatitis in the pediatric population 2, 4, 5, 6.

Children undergoing treatment for hemato‐oncological diseases comprises a specific patient population with certain characteristic in common: they have systemic disease, they are undergoing treatment with chemotherapeutics, they are immunocompromised, they have more surgical procedures under general anesthesia and they frequently receive antimicrobial treatment. This puts them at greater risk of developing AP. Currently, there are no published reviews on AP in this specific group of pediatric patients.

The purpose of this review was to present a systematic account of the various etiological factors for developing AP during childhood cancer treatment and to highlight modern trends in the diagnosis and treatment of AP in the pediatric population.

## Background

### Etiology

Various etiologies have to be considered when AP is suspected in children (Table 1) 7; however, some etiologies are more frequent then others in oncological pediatric patients as described below.

**Table 1:** Etiologies of pancreatitis in children and adolescents

Systemic disease	Cystic fibrosis, Crohns disease, rheumatoid arthritis, hemolytic‐uremic syndrome, diabetes mellitus, systemic lupus erythematous, etc.
Abdominal trauma	Bicycle/car‐accidents, child abuse, sports injuries
Biliary disease	Gallstones, sludge, choledochuscysts, cholangitis
Structural	Pancreas divisum/annulare, common channel syndrome, residual condition after duodenal and pancreatic surgery, duodenal diverticulum, and duodenal duplication
Infections	EBV, Enterovirus, Salmonella, Mononucleosis, Mumps, Mycoplasma, Kawasaki, Coxsackie, Ascaris, Candida, etc.
Medications	L‐Asparaginase, 6‐mercaptopurine, pentamidine, valproic acid, furosemid, 5‐ASA/Salazopyrin, tetracyklins, prednisone, etc.
Metabolic	Hypertriglyceridemia, hypercalcemia, alfa 1‐antitrypsinbrist, diabetic ketoacidosis, etc.
Genetic (hereditary)	PRSS‐1‐, SPINK‐1‐, CFTR‐, and CFTR‐mutations
Autoimmune pancreatitis	
Idiopathic	In some studies up to a third of pediatric patients no cause is determined
Others	Transplantation particularly liver and bone marrow, post‐ERCP and pancreatic, tumors (ex pseudocysts, pancreatoblastoma, and solid pseudopapillary tumor)

EBV, Epstein–Barr virus; 5‐ASA, 5‐aminosalicylic acid; SPINK‐1, serine protease inhibitor, Kazal type‐1; CFTR, cystic fibrosis transmembrane conductance regulator; ERCP, endoscopic retrograde cholangiopancreatography.

#### Drugs

Numerous medications causing drug‐induced pancreatitis have been indicated in the literature 8. Although some have been reported to cause severe pancreatitis, it is important to stress that the etiology for AP does not determine its severity 9. Once the process of AP has been initiated, its severity is determined by the propagation of proinflammatory mediators, like IL‐1*β*, IL‐6, and TNF‐*α* 10.

L‐Asparaginase is the key chemotherapeutic agent used for remission induction and consolidation therapy in acute lymphoblastic leukemia (ALL) and it is also an important component of the therapy for some non‐Hodgkin lymphomas (NHL) 11, 12. Cases of L‐asparaginase‐associated pancreatitis (AAP) were first reported in the 1970s with an incidence ranging from 0.7% to 24% 13, 14, 15, 16 and mortality rates of 2–5% 14. In a retrospective study by Knoderer et al. of 254 patients receiving L‐asparaginase, 48 patients developed pancreatitis, of whom 33 (68.7%) were indicated as having AAP; however, the authors did not succeed in predicting which patients would develop pancreatitis. All 33 patients with AAP had mild cases of pancreatitis 11. And other authors have also reported that patients with AAP presented with mild symptoms 14, 15. In contrast, several other case reports have described various complications associated with AP 17, 18, 19, 20, some with fatal results 21. Chronic complications rarely occur, but when they do, they can take the form of chronic pancreatitis or diabetes mellitus 16. AAP has been reported after treatment with all three asparaginase formulations 11, 12, 13 and Alvarez et al. suggested that the incidence of PEG‐asparaginase‐induced pancreatitis was higher than that induced by *E. coli*‐asparaginase (18% vs. 1.9%) 12. However, Kearney and colleagues reported that, in a cohort of 403 patients, the only predisposing factor for developing AAP was age, as older children had a higher risk of developing the disease. Also, the patients who experienced AAP had a significantly higher risk of a relapse of their cancer 14. In addition to asparaginase, other chemotherapeutic agents have also been indicated in the literature as possible causes for inducing pancreatitis (Table 2).

**Table 2:** Frequently used drugs during childhood cancer treatment that are implicated in AP etiology

Drug group	Drug	Classification by Trivedi et al. 8	Classification by Badalov et al. 29	Other references
Chemotherapeutics	L ‐ Asparaginase	Class I	Class II	11, 12, 13, 14, 15, 17, 18, 19, 20, 21
	Mercaptopurine	Class I	Class Ib	22, 23, 24
	Cytosine arabinoside	Class I	Class Ib	25, 26, 27, 28
	Ifosfamide	/	Class Ib	30, 31
	Cisplatin	Class II	/	32
	Doxorubicin	Class III	/	
	Methotrexate	Class III	/	33
Immunomodulators	Tacrolimus	Class III	Class IV	47, 48
	Cyclosporine	Class III	Class III	49
Steroids	Steroids	Class I		34, 35
	Dexamethasone		Class Ib	34
	Prednisone		Implicated as causing AP	34
Antimicrobial drugs	TMP/SMX	Class I	Class Ia	11, 36, 37
	Erythromycin	Class II	Class II	
	Tetracyclines	Class I	Class Ia	
	Cidofovir	Class III	/	
	Ganciclovir	Class III	/	
	Ribavirine	Class III	/	
	Voriconazole	Class III	/	
Other drugs	Propofol	Class III	Class II	38, 39, 40
	Omeprazole	Class III	Class Ib	41, 42
	Paracetamol	/	/	43, 44
	Furosemide	Class I	Class Ib	

TMP/SMX, trimethoprim/sulfomethoxazole; AP, acute pancreatitis.

Steroids are known to cause pancreatitis when used in the therapy of malignant disease as well as other medical conditions. In a meta‐analysis comparing the corticosteroids, dexamethasone, and prednisone, used for induction therapy in childhood ALL, Teuffel et al. found that the risk ratio for AP induced by these two corticosteroids was 3.78% and that there was no significant difference between the two drugs 34. Bai et al. reported that corticosteroids, along with valproic acid, were the two drugs most associated with drug‐induced pancreatitis in children 35.

Due to frequent episodes of febrile neutropenia, sepsis and other bacterial, viral and fungal infections during chemotherapy, antimicrobial agents are frequently used. Those associated with AP are listed in Table 2. The most controversial are trimethoprim/sulfomethoxazole (TMP/SMX) since this combination is used universally in patients undergoing chemotherapy as prophylaxis against *Pneumocystis jirovecii* infection. It has been noted as the drug associated with AP in many studies, with reported recurrence of AP after rechallenge, although this is mostly seen in adults 29, 36, 37. However, Knoderer et al. did not observe a role for TMP/SMX in causing AP in a pediatric ALL population 11. No controlled study has been performed to examine the role of TMP/SMX in the pathogenesis of AP; it is possible that the pathogenesis depends on the dosage.

Certain other drugs, probably used more frequently in hemato‐oncological patients than in the general patient population, have been listed as possible causes of AP (Table 2). Propofol, which is the most frequently used anesthetic, is reported to cause AP 8, 29, 38, 39. However, in a retrospective study in which five of 479 children receiving chemotherapy for ALL developed AP, Crawford et al. reported that no episode of AP occurred within the latency period of propofol and that all five patients received propofol anesthesia after episodes of AP without complications 40. In our experience, it seems that episodes of AP in hemato‐oncological pediatric patients are unlikely caused by propofol.

During corticosteroid therapy most children need proton pump inhibitors (PPIs), such as omeprazole, which is reported to cause AP in the adult population. In addition, other PPIs and antacids such as cimetidine and ranitidine are also reported as possible causes of AP 8, 29, 41. Eland et al. conducted a retrospective cohort study in which they concluded that there is no association between AP and the use of acid‐suppressing drugs 42. However, there is no published data from the pediatric population to support this claim.

#### Transplantation

Hematopoietic stem cell transplantation (HSCT) is an important means of treating some hemato‐oncological diseases. AP was first recognized as a possible complication of HSCT in the early 1990s 44. Nowadays, pancreatitis is well recognized as a complication of HSCT and it has been linked to many factors involved in the transplantation procedure. Werlin et al. reported the incidence of AP as 3.5% among 202 children treated with HSCT 45 with a similar incidence of post‐HSCT pancreatitis (7 of 180 or 3.9%) in a pediatric population with pancreatitis due to various etiologies seen during a six‐year period 4. Ko et al. found pancreatitis at autopsy in 28% of patients treated with HSCT (51 of 184), and in five of these, pancreatitis was the cause of death. An increase in the number of survival days and any graft‐versus‐host disease (GVHD) found at autopsy were cited as risk factors for developing AP in these patients 46.

Some case reports have shown that drugs used in the stem cell transplantation process are also possible causes of AP 47, 48, for example, the immunosuppressive drugs, cyclosporine and tacrolimus, and the antiviral drugs, cidofovir, foscarnet, and ganciclovir, have all been associated with pancreatitis 8, 29. Using a rat model, Ito et al. showed that therapeutically recommended doses of cyclosporine can induce AP 49.

There is also some evidence that infection with cytomegalovirus (CMV) or adenovirus can cause AP 50, 51. Bateman et al. reported that five out 95 (5,3%) patients treated with HSCT experienced pancreatitis, one following treatment with tacrolimus 47 and four after adenoviral infection 52. In addition, a mouse model has been used to show that adenovirus infection can cause AP 53. Salomone et al. suggested that in patients with post‐HSCT complications, particularly acute hepatic/hepatointestinal GVHD and CMV infection, the possibility of AP should be considered 54. The reactivation of the varicella‐zoster virus, which is likely to occur in post‐HSCT patients, has also been described as a possible cause of AP 55, 56.

### Diagnostics

Acute pancreatitis should always be included in the differential diagnosis of a child presenting with abdominal pain during cancer treatment!

The definition and diagnostic criteria for pediatric AP given by the INSPPIRE Project (International Study Group of Pediatric Pancreatitis: In Search for a Cure) are outlined in Table 3 57.

**Table 3:** Clinical definition of AP in children 7

	Requires at least 2 of 3 criteria
Acute pancreatitis (AP)	1 Abdominal pain suggestive of, or compatible with AP (i.e., abdominal pain of acute onset, especially in the epigastric region)
	2 Serum amylase and/or lipase activity at least three times greater than the upper limit of normal (international units/liter)
	3 Imaging findings characteristic of, or compatible with AP (e.g., using US, CECT, EUS, MRI/MRCP)

US, transabdominal ultrasonography; CECT, contrast‐enhanced computerized tomography; EUS, endoscopic ultrasonography; MRI/MRCP, magnetic resonance imaging/magnetic resonance cholangiopancreatography.

Abdominal pain in children has variable characteristics but is still the most common symptom of AP, occurring in 87% of cases, followed by vomiting, abdominal distress and tenderness 58, thus AP should be strongly suspected when sudden abdominal pain is accompanied by nausea and vomiting. Generally, levels of amylase and lipase elevated to three times the normal levels will confirm the diagnosis. Since the simultaneous elevation of both pancreatic enzymes in pediatric patients increases the sensitivity of the test to 94%, the analysis of both enzymes is recommended, especially in very young children 1, 58, 59, 60, 61. Table 4 illustrates the main nonpancreatic causes of increased pancreatic enzyme levels. Additionally, in a population of adult patients, it has been demonstrated that an elevated creatinine level during a 48‐hour period, despite adequate hydration, is associated with the development of pancreatic necrosis with a positive predictive value of 93% 62.

**Table 4:** Main nonpancreatic causes of increased pancreatic enzyme levels 1, 2

Amylase		Lipase
Abdominal causes	Nonabdominal causes	
Biliary tract disease Intestinal obstruction/ischemia Mesenteric infarction Peptic ulcer Appendicitis Pancreatic cancer Ruptured ectopic pregnancy Prostate disease Ovarian neoplasm Afferent loop obstruction Dissecting aortic aneurysm	**Salivary gland**Salivary traumaInfection (mumps)Salivary duct obstruction***Irradiation*** **Thoracic**Myocardial infarctionPulmonary embolismPneumoniaMetastatic lung cancerCardiopulmonary bypass**Metabolic**Diabetic ketoacidosis**Drugs**OpiatesPhenylbutazone**Trauma**Cerebral traumaBurns**Renal disease**Renal insufficiencyRenal transplantation**Macroamylasemia**	Pancreatic cancerNonpancreatic abdominal painMacrolipasemiaRenal insufficiencyAcute cholecystitisEsophagitisHypertriglyceridemia

In addition to clinical symptoms and laboratory tests, diagnostic imaging plays a critical role in the evaluation of AP by determining its severity and identifying potential complications 2, 63. The recommended imaging modalities in pediatric population are similar to those for adult patients, but pediatric experts are more likely to select tests that limit radiation and recognize that certain modalities may be more difficult to perform because of patient size and sedation need 57.

Abdominal ultrasound (US) and computed tomography (CT) scans remain the most commonly used imaging methods. US is performed most frequently due to its simplicity and the absence of radiation; however, about one‐third of US findings are normal 11, 12. According to UK guidelines it is not current practice to perform early CT scans for the detection and staging of severe cases of AP, due to its low sensitivity 64. On the other hand, Tsuji et al. showed that perfusion CT was superior to angiography in predicting pancreatic necrosis in early SAP and that its sensitivity and specificity were 100% and 95.3%, respectively 65, 66, 67: it is also a useful tool for predicting systemic complications 67.

Magnetic resonance imaging associated with cholangiopancreatography seems to be an excellent alternative in the evaluation of the pancreas, due to the absence of radiation and lack of invasiveness 59.

Finally, when infected pancreatic necrosis is suspected, fine‐needle aspiration is recommended as a diagnostic procedure for identification of the pathogen with a possibility of making the right diagnosis in 89–100% of cases 68.

### Complications

In the majority of AP cases the course of the disease is mild and self‐limiting and complications are relatively rare. For example, Knoderer et al. reported that 43 patients of 48 who developed AP during treatment for ALL, acute myeloid leukemia (AML), or NHL were classified as mild cases and complications such as necrosis or pseudocysts documented by imaging methods, were present in only 10% of patients 11. Kearney et al. reported that only 18% of patients with AAP developed complications such as pseudocysts 14.

The complications observed in children with AP can be immediate or delayed. Immediate complications include hypovolemic and septic shock, renal dysfunction, cavity effusions, and acute respiratory distress syndrome. The most common delayed complications include pancreatic necrosis and the formation of pseudocysts 1, 59. Pancreatic necrosis has an infection rate of between 30% and 70%, and the immediate identification of this complication is critical for the child's prognosis since it often progresses to multisystem failure 59. It has been shown that 80% of all AP deaths are due to infected necrosis of the pancreas and consequently to septic complications 69, 70. With respect to pseudocysts, there are numerous case reports showing pseudocyst development during cancer treatment in children, most of them during L‐asparaginase treatment 18, 19, 20, 71.

### Treatment

While the etiology of childhood AP differs from AP in adult patients, its treatment, like its diagnostics, is based on current adult therapy strategies. Since there is no specific treatment for AP, various different guidelines have been published and these are not always in agreement 64, 72, 73. For example, administration of prophylactic antibiotics for the prevention of pancreatic infection in SAP is contentious. Two controlled, double‐blind studies were published: Isenmann et al. 74 using ciprofloxacin/metronidazole and Garcia‐Barrasa et al. 70 using ciprofloxacin, both showing no difference in mortality or the incidence of pancreatic infection between placebo and the studied medication groups. In contrast, regarding necrotizing AP, two separate meta‐analyses concluded that antibiotic prophylaxis is superior to antibiotic treatment on demand and that the patients with proven pancreatic necrosis should receive either imipenem or meropenem prophylaxis 69, 75. On the contrary, a third meta‐analysis performed in 2008 concluded that the use of prophylactic antibiotics for acute necrotizing pancreatitis had no effect on infected pancreatic necrosis and no effect on the mortality rate among these patients 76.

The use of protease inhibitors, applied either intravenously or by continuous regional arterial infusion (CRAI), to treat patients with AP remains controversial and is recommended only in Japanese (JPN) and Italian national guidelines 73, 77. Morimoto et al. published a report describing five pediatric patients with severe AAP who were successfully treated using CRAI with protease inhibitors and antibiotics resulting in a swift resumption of chemotherapy and allowing the patients to remain in complete remission with further chemotherapy that excluded L‐asparaginase 78.

Despite initially encouraging results in large randomized studies, antisecretory agents such as octreotide 79, and anti‐inflammatory drugs such as lexipafant, have proved disappointing as therapies for AP 64, 77. On the other hand, more recent studies report on the use of ocreotide as a means of preventing AP during L‐asparaginase readministration 80.

However, the available guidelines do agree on other areas of AP treatment 64, 72, 73. At the onset of symptoms, and before the disease severity is known, supportive care is the most important factor. Critical components in the care of patients with AP include oxygen supplementation and fluid resuscitation. Although pain control is mentioned only in the JPN guidelines, it is crucial during the care of a patient with AP. Currently, enteral rather than total parenteral nutrition is recommended in cases where nutrition support is necessary. The current data do not support nasogastric suction, gut decontamination or the use of H2 blockers in AP, unless other indications are present. Furthermore, all patients with predicted SAP should be monitored closely and prompt transfer to an intensive care unit should take place in the case of sustained organ failure.

Opinions regarding the treatment of infected pancreatic necrosis are generally in accord in the current literature and the treatment of choice is surgical debridement. As an alternative, minimally invasive approaches may be used in selected circumstances with the appropriate expertise. On the other hand, the approach for sterile necrosis is conservative at the onset of the disease, and if surgical treatment is needed it should be delayed as long as possible. Debridement should only be considered if abdominal pain persists and prevents oral intake. Conservative treatment is also recommended for pancreatic pseudocysts but occasionally a patient may require surgical, endoscopic or radiological intervention. The condition should be managed on an individual basis according to how long the cyst has persisted, its symptoms, accompanying complications or an increase in diameter.

At the Karolinska University Hospital the initial treatment of AP consists of “pancreatic rest” by fasting followed by limited pancreatic stimulation, pain management, antiemetics, intravenous fluid therapy, and careful monitoring for early detection and treatment of possible complications. It is also important to determine the etiology of the pancreatitis in order to determine whether the cause itself is treatable. Upon admission, the patient is usually rehydrated (12.5 mL/kg/h, for 4 h), and this is often followed by fluid maintenance 120–150% of the continuous basal rate. This fluid therapy is managed on an individual basis and is based on objective measurements and the monitoring of electrolytes and fluid balance aiming for a urine output of at least 0.5–1 mL/kg per hour). In most cases of AP enteral nutrition, which stabilizes the gut barrier, stimulates the motility and prevents the overgrowth of bacteria, can be introduced within 48 hours. At Karolinska University hospital, we have great experience with nasojejunal tubes as an enteral route for feeding in more complicated cases, were oral nutrition or nasogastric tube have failed. Parenteral nutrition is rarely required, though it could be necessary if longer or more complicated episodes of pancreatitis occur. Although certain adult studies have shown increased risk of complications, such as infections 81 and mortality 82 with parenteral nutrition, it is still used in over 40% of the pediatric patients 58.

Pain management is important for comfort and for reducing energy expenditure. To achieve this, the intravenous administration of analgesics such as morphine or other opioids and nonopioid drugs, such as paracetamol, is often required. In the INSPPIRE study 56, 94% of the pediatricians used opioids and it seems safe to use them without worsening the pancreatitis by inducing spasms in the sphincter of Oddi 83. With respect to the management of infection, at the Karolinska University Hospital, antibiotics are administrated only if sepsis, concomitant bacterial infection or infected necrotizing pancreatitis occurs. Therapeutic pediatric endoscopic retrograde cholangiopancreatography may be required in complicated cases involving duct‐rupture, main pancreatic duct (MPD) stones, or in pancreatic pseudocysts that compress the MPD. Some cases of AP with severe complications due to chemotherapy agents need repeated treatment with endoscopic transgastric and/or transpappillary stents. Such procedures are best performed with the patient under general anesthesia, which provides a good opportunity for introducing a nasogastric jejunal tube beyond the ligament of Treitz. In very rare and complicated cases of AP invasive surgery is needed.

#### What type of aftercare?

One of the main questions when childhood cancer treatment is complicated with AP is what course of treatment to follow after an episode of AP, especially when it is drug‐related. When there is an adequate replacement for an AP‐inducing drug it should be considered. Sastry et al. reported replacing tacrolimus with cyclosporine in a patient with tacrolimus‐induced AP without recurrence of AP symptoms 47. In general, the main uncertainty reported in the current literature is the question of whether or not to restart L‐asparaginase treatment after an episode of AAP, as L‐asparaginase is an important drug for remission maintenance in ALL treatment. Secondary episodes of AP after L‐asparaginase re‐challenge have been reported but these generally occur without life‐threatening complications 11, 14. There have also been reported cases of patients that stayed in remission even when L‐asparaginase was omitted from chemotherapy 19, 77; however, other cases have been reported of patients who relapsed and died 19. The main factors affecting the decision of whether or not to restart L‐asparaginase treatment include the time of AP onset during chemotherapy and the severity of the episode of AP.

Wu et al. reported replacing L‐asparaginase with methotrexate after AP. In this study, four patients, treated with octreotide for AP, stayed in remission and one, who did not receive octreotide, died due to relapse of ALL 79. In contrast, Hung et al. reported a case of ifosfamide‐induced AP during treatment of osteosarcoma in which a second episode of AP occurred after ifosfamide re‐challenge; however, the symptoms of AP were resolved and the patient stayed in remission 31. Therefore, until the predisposing factors for AP development have been identified, cases reported in the literature suggest that one episode of mild pancreatitis may not be an absolute contraindication to the administration of further doses of the chemotherapeutic agent suspected of causing the AP 14. For clinicians, it seems essential to find a balance for each separate case between the risk of AP recurrence and the potential therapeutic benefits of restarting a chemotherapeutic agent that is potentially vital in ensuring that the patient stays in remission.

## Conclusions

The risk of developing AP during childhood cancer treatment is significant; therefore, it is of critical importance to be able to recognize the symptoms of AP, and to try, if possible, to determine its etiology and to treat the disease in the best possible way according to its severity, complications and using the available guidelines. Fast resolution of AP symptoms and continuation with chemotherapy is vital to achieve remission of the underlying malignant disease.

## Conflict of Interest

None declared.
